# Clinical features of asthma with connective tissue diseases

**DOI:** 10.1111/crj.13595

**Published:** 2023-02-18

**Authors:** Keisuke Watanabe, Nobuyuki Horita, Yu Hara, Nobuaki Kobayashi, Takeshi Kaneko

**Affiliations:** ^1^ Department of Pulmonology Yokohama City University Graduate School of Medicine Yokohama Japan

**Keywords:** asthma, connective tissue diseases, lung function, rheumatoid arthritis, T2‐low asthma

## Abstract

**Background:**

The clinical features of asthma with connective tissue diseases (CTDs) are not well‐known. This study therefore aimed to investigate the clinical characteristics of asthma with CTDs.

**Methods:**

We retrospectively examined the records of adults (≥18 years old) with asthma followed up between January 2010 and December 2019. We then compared the clinical features of asthma with and without CTDs.

**Results:**

Among 568 subjects with asthma, 42 subjects (7.4%) had CTDs. The most frequent concomitant CTD was rheumatoid arthritis (*n* = 23, 54.8%), followed by systemic lupus erythematosus (*n* = 6, 14.3%). The proportion of women (with vs. without CTDs, 85.7% vs. 56.5%, *p* < 0.001) and Global Initiative for Asthma step were higher (Step 4 or 5, with vs. without CTDs, 81.0% vs. 62.0%, *p* = 0.01) in asthma with CTDs, whereas frequency of allergic rhinitis was higher in asthma without CTDs (with vs. without CTDs, 7.1% vs. 26.1%, *p* = 0.005). Eosinophil ratio (with vs. without CTDs, 2.1% vs. 3.5%, *p* = 0.009) and total immunoglobulin E level (with vs. without CTDs, 43 IU/mL vs. 237 IU/mL, *p* = 0.002) were lower in asthma with CTDs. In terms of lung function, percentage predicted forced vital capacity (with vs. without CTDs, 86.7% vs. 99.7%, *p* = 0.008) and percentage predicted forced expiratory volume in 1 s (%FEV1) (with vs. without CTDs, 77.2% vs. 88.4%, *p* = 0.02) were all lower in asthma with CTDs. With multivariable analysis, CTDs (odds ratio [OR] 2.8, 95%CI 1.3–6.0; *p* = 0.008), chronic obstructive pulmonary disease (OR 3.8, 95%CI 2.1–6.7; *p* < 0.001) and asthma onset at <20 years old (OR 1.8, 95%CI 1.1–3.2; *p* = 0.03) were associated with low FEV1 (defined as %FEV1 < 80%) in asthma.

**Conclusions:**

Asthma with CTDs was related to lower lung function and low‐T2 inflammation asthma.

## Graphical abstract

The aim of this study was to investigate the clinical characteristics of asthma with connective tissue diseases. Asthma with connective tissue diseases was related to lower lung function and low‐T2 inflammation asthma.

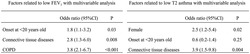

## 1 INTRODUCTION

Asthma is characterized by chronic airway inflammation, airway hypersensitivity and reversible airway obstruction. More than 330 million people around the world suffer from asthma,^1^ and individuals with asthma can show a variety of comorbidities.^2^, ^3^ Some asthma comorbidities have been studied in detail, including allergic rhinitis (AR), sinusitis, chronic obstructive pulmonary disease (COPD) and obesity. In particular, COPD (asthma–COPD overlap: ACO)^4^ and upper airway disease (AR and sinusitis; one airway, one disease)^5^ have been intensively researched and are well‐recognized comorbidities. Recently, relationships between asthma and connective tissue diseases (CTDs) have been reported^6^, ^7^, ^8^, ^9^, ^10^, ^11^ but remain relatively poorly characterized. Individuals with asthma have higher risks of rheumatoid arthritis (RA), systemic lupus erythematosus (SLE) and other collagen diseases.^6^, ^8^, ^9^, ^10^, ^11^ Conversely, an increased risk of asthma has been reported among individuals with RA.^7^ Luo et al. reported RA as a risk factor for in‐hospital mortality during asthma exacerbations.^12^ However, the clinical features of asthma with CTDs are not well‐known. The aim of this study was therefore to investigate the clinical characteristics of asthma with CTDs.

## 2 PATIENTS AND METHODS

We retrospectively examined the records of adults (≥18 years old) with physician‐diagnosed asthma who had been followed up for >1 year at our hospital between January 2010 and December 2019 (also defined as the study period). All subjects had been diagnosed by a pulmonologist and/or allergist at our hospital. To confirm the diagnosis of asthma, we only included subjects with follow‐up >1 year. We collected data from spirometry, peripheral eosinophil counts and total immunoglobulin E (IgE) during a stable state of asthma within 2 years before or after the first visit of during the study period. We also collected findings from chest computed tomography (CT) if the subject underwent CT within 2 years before and/or after the start of the study period. Specific IgE status at diagnosis and/or during follow‐up was also collected. We then compared clinical features between asthma with and without CTDs.

CTDs were diagnosed by rheumatologists independent of our study. We excluded vasculitides (anti‐neutrophilic cytoplasmic antibody‐related vasculitis and Behçet disease) and IgG4‐related diseases from the investigated CTDs.

Because there is no consensus diagnostic criteria of COPD in asthma (ACO),^13^, ^14^ we made the diagnosis of concomitant of ACO with post‐bronchodilator FEV1/FVC < 0.7 and smoking history of ≥10 pack‐year in ≥40‐year‐old subjects. Smoking history of ≥10 pack‐year in ≥40‐year‐old was chosen based on previous studies.^13^, ^14^

Data are presented as median (range) unless otherwise specified. JMP version 11 software (SAS Institute, Cary, NC, USA) was used for all statistical analyses. Comparisons were made using *t*‐tests or the Mann–Whitney *U* test for continuous variables. Categorical variables were compared using Pearson's chi‐square test or Fisher's exact test. To detect whether CTDs is an independent risk factor for low FEV1 [percentage predicted forced expiratory volume in 1 s (%FEV1) < 80%] and low‐T2 asthma, we performed multivariate analysis by logistic regression analysis for factors showing values of *p* < 0.1 in univariate analysis. Among the subjects who had the data of both eosinophils in peripheral blood and IgE, high‐T2 asthma was defined as having eosinophils in peripheral blood ≥300 cells/μL and/or IgE ≥ 75 IU/mL.^15^ Low‐T2 asthma was defined as having eosinophils in peripheral blood <300 cells/μL and IgE < 75 IU/mL. Statistical significance was set for values of *p* < 0.05, and all tests were two‐tailed.

This study was approved by the institutional review board of Yokohama City University Hospital (approval no. F210900006). Due to the retrospective nature of this study, the need to obtain written informed consent was waived by the institutional review board of Yokohama City University Hospital. All methods were carried out in accordance with relevant guidelines and regulations.

## 3 RESULTS

**FIGURE 1:**
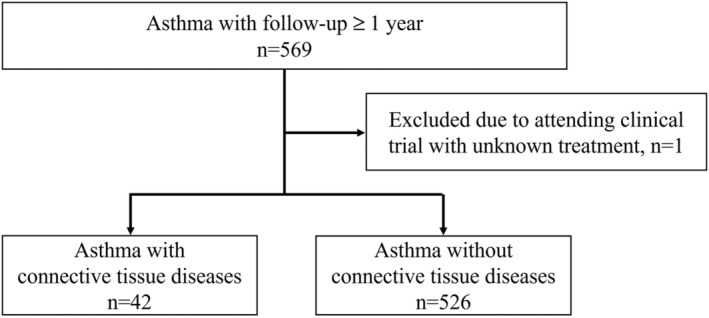
Flow diagram for participation in the study.

We detected 569 patients with asthma who were followed up for >1 year. One patient was excluded from the study because of attending a clinical trial involving unknown treatment. As a result, 568 subjects were included in this study, including 42 subjects (7.4%) with one or more CTDs (Figure 1).

Table 1 shows underlying CTDs. The most common concomitant CTD was RA (*n* = 23, 54.8%), followed by SLE (*n* = 6, 14.3%). Some subjects showed multiple CTDs (Table 1).

**TABLE 1:** Main underlying CTD (*n* = 42).

	*n* (%)
Rheumatoid arthritis	23 (54.8)
Systemic lupus erythematosus	6 (14.3)
Sjögren syndrome	3 (7.1)
Systemic sclerosis	3 (7.1)
Dermatomyositis/polymyositis	2 (4.8)
Others	5 (11.9)

*Note*: Two subjects had both rheumatoid arthritis and Sjögren syndrome, one subject had both systemic lupus erythematosus and Sjögren syndrome and one subject had both systemic sclerosis and Sjögren syndrome. Diagnoses in ‘others’ included mixed connective tissue disease, undifferentiated connective tissue disease, adult Still's disease, spondyloarthritis and remitting seronegative symmetrical synovitis with pitting edema, in one subject each.

Table 2 shows the clinical features of asthma with or without CTD. The proportion of women (36/42 [85.7%] vs. 297/526 [56.5%], *p* < 0.001), proportion using a long‐acting β2‐agonist (LABA) (88.1% vs. 65.0%, *p* = 0.002) and Global Initiative for Asthma (GINA) step were all higher in asthma with CTDs, whereas the frequency of AR was higher in asthma without CTDs (7.1% vs. 26.1%, *p* = 0.005).

**TABLE 2:** Clinical features of asthma with or without CTDs.

	Asthma with CTDs (*n* = 42)	Asthma without CTDs (*n* = 526)	*p*
Age (years)	62 (36–82)	60 (21–88)	0.16
Sex (male/female)	6/36	229/297	<0.001
Smoking history (never/ex/current)	30/8/3 (*n* = 41)	277/165/69 (*n* = 511)	0.07
GINA Step 4 or 5	34 (81.0%)	326 (62.0%)	0.01
Medication			
ICS	39 (92.9%)	500 (95.1%)	0.47
LABA	37 (88.1%)	342 (65.0%)	0.002
LAMA	8 (19.1%)	47 (8.9%)	0.05
LTRA	13 (31.0%)	174 (33.1%)	0.87
Theo	11 (26.2%)	108 (20.5%)	0.43
Maintenance OCS	3 (7.1%)	18 (3.4%)	0.20
Onset at <20 years old	7 (17.5%) (*n* = 40)	114 (23.4%) (*n* = 487)	0.56
Allergic asthma	14 (33.3%)	241 (45.8%)	0.15
Comorbidities			
COPD	4 (9.5%) (*n* = 42)	69 (14.3%) (*n* = 482)	0.49
AR	3 (7.1%)	137 (26.1%)	0.005
Sinusitis	2 (4.8%)	59 (11.2%)	0.30

*Note*: Data are presented as median (range) or number of patients (%). GINA treatment step and medication are presented as of the start of the study period. OCS used for reasons other than asthma was not included in evaluation of the treatment step of asthma. Allergic asthma was defined as at least one positive result for specific immunoglobulin E and/or symptom with exposure to allergens. Inhaled allergens associated with asthma are listed in the Supporting Information.

Abbreviations: AR, allergic rhinitis; COPD, chronic obstructive pulmonary disease; GINA, Global Initiative for Asthma; ICS, inhaled corticosteroid; LABA, long‐acting β2‐agonist; LAMA, long‐acting muscarinic agent; LTRA, leukotriene receptor antagonist; OCS, oral corticosteroid; Theo, theophylline.

Table 3 shows the laboratory findings of asthma with or without CTDs. Eosinophil ratio (2.1% vs. 3.5%, *p* = 0.009) and total IgE level (43 IU/mL vs. 237 IU/mL, *p* = 0.002) were lower in asthma with CTDs. In terms of lung function, percentage predicted forced expiratory volume in 1 s (FEV1) (%FEV1) (77.2% vs. 88.4%, *p* = 0.02) was lower in asthma with CTDs. But, percentage predicted forced vital capacity (FVC) (%FVC) was lower in asthma with CTDs, FEV1/FVC was not significantly different among asthma with or without CTDs (Table 3).

**TABLE 3:** Laboratory findings of asthma with or without CTDs.

	Asthma with CTDs (*n* = 42)	Asthma without CTDs (*n* = 526)	*p*
Eosinophils in peripheral blood	*n* = 40	*n* = 465	
Eosinophil ratio (%)	2.1 (0.2–16.1)	3.5 (0–33.4)	0.009
Absolute number (cells/μL)	151 (15–890)	219 (0–2347)	0.06
Immunoglobulin E (IU/mL)	43 (1–852) (*n* = 24)	237 (1–81 189) (*n* = 260)	0.002
Lung function	*n* = 34	*n* = 329	
FVC (L)	2.17 (1.13–4.57)	2.84 (0.95–6.48)	<0.001
FVC % predicted (%)	86.7 (54.5–127.7)	99.7 (25.0–161.1)	0.007
FEV1 (L)	1.41 (0.67–2.39)	2.01 (0.48–4.37)	<0.001
FEV1/FVC (%)	70.7 (33.2–92.1)	71.7 (27.6–94.5)	0.58
FEV1% predicted (%)	77.2 (36.8–105.7)	88.4 (21–112.2)	0.02

*Note*: Data are presented as median (range).

Abbreviations: FEV1, forced expiratory volume in 1 s; FVC, forced vital capacity.

Table 4 and Table 5 show factors related to low FEV1 in asthma according to univariate and multivariable analysis. CTDs (odds ratio [OR] 2.8, 95%CI 1.3–6.0; *p* = 0.008), COPD (OR 3.8, 95%CI 2.1–6.7; *p* < 0.001) and asthma onset at <20 years old (OR 1.8, 95%CI 1.1–3.2; *p* = 0.03) were independently associated with low FEV1 in asthma. Because COPD cause low FEV1, we analysed the factors related to low FEV1 in asthma without COPD (Table S1). CTDs were independently associated with low FEV1 in asthma without COPD (OR 2.2, 95%CI 1.1–4.5; *p* = 0.03).

**TABLE 4:** Factors related to low FEV1 in asthma.

	Asthma with low FEV1 (*n* = 129)	Asthma without low FEV1 (*n* = 235)	*p*
Age (years)	63 (22–84)	61 (21–88)	0.38
Sex (male/female)	60/69	89/146	0.12
Smoking history (never/ex/current)	66/45/17 (*n* = 128)	135/70/29 (*n* = 234)	0.52
Onset at <20 years old	32 (26.9%) (*n* = 119)	39 (17.6%) (*n* = 222)	0.05
Allergic asthma	59 (45.7%)	100 (42.6%)	0.58
CTDs	18 (14.0%)	16 (6.8%)	0.04
COPD	41 (31.8%) (*n* = 129)	25 (10.7%) (*n* = 233)	<0.001
AR	28 (21.7%)	60 (25.5%)	0.45
Sinusitis	16 (12.4%)	28 (11.9%)	0.87

Data are presented as median (range) or number of patients (%).

Low FEV1 is defined as percentage predicted forced expiratory volume in 1 s (%FEV1) < 80%.

AR, allergic rhinitis; COPD, chronic obstructive pulmonary disease; CTDs, connective tissue diseases.

**TABLE 5:** Factors related to low FEV1 with multivariable analysis.

	Odds ratio (95%CI)	P
Onset at <20 years old	1.8 (1.1–3.2)	0.03
CTDs	2.8 (1.3–6.0)	0.008
COPD	3.8 (2.1–6.7)	<0.001

Low FEV1 is defined as percentage predicted forced expiratory volume in 1 s (%FEV1) < 80%.

COPD, chronic obstructive pulmonary disease; CTDs, connective tissue diseases.

Table 6 and Table 7 show factors related to low‐T2 asthma according to univariate and multivariable analysis. CTDs (odds ratio [OR] 3.9, 95%CI 1.5–9.8; *p* = 0.004) and female (OR 2.5, 95%CI 1.2–5.4; *p* = 0.02) were independently associated with low‐T2 asthma.

**TABLE 6:** Factors related to low‐T2 asthma.

	Low‐T2 asthma (*n* = 53)	High‐T2 asthma (*n* = 223)	*p*
Age (years)	62 (22–85)	57 (21–85)	0.19
Sex (male/female)	12/41	98/125	0.005
Smoking history (never/ex/current)	30/17/6 (*n* = 53)	111/79/28 (*n* = 218)	0.76
Onset at <20 years old	7 (14.0%) (*n* = 50)	53 (25.2%) (*n* = 210)	0.096
Use of systemic steroids other than asthma	17 (32.1)	54 (24.2%)	0.29
CTDs	11 (20.8%)	12 (5.4%)	0.001
COPD	4 (7.7%) (*n* = 52)	33 (15.7%) (*n* = 210)	0.18
AR	13 (24.5%)	62 (27.8%)	0.73
Sinusitis	6 (11.3%)	31 (13.9%)	0.82

*Note*: Data are presented as median (range) or number of patients (%).

Abbreviations: AR, allergic rhinitis; COPD, chronic obstructive pulmonary disease; CTDs, connective tissue diseases.

**TABLE 7:** Factors related to low‐T2 asthma with multivariable analysis.

	Odds ratio (95%CI)	*p*
Female	2.5 (1.2–5.4)	0.02
Onset at <20 years old	0.6 (0.2–1.4)	0.25
CTDs	3.9 (1.5–9.8)	0.004

Abbreviation: CTDs, connective tissue diseases.

Table S2 shows findings from chest CT in asthma with CTDs. Of the 42 subjects, 36 subjects underwent chest CT within 2 years before or after the start of the study period. Bronchiolectasis (BE) was the most common CT finding (*n* = 10, 27.8%) in this study.

## 4 DISCUSSION

This study investigated the clinical features of asthma with CTDs. Patients with asthma and CTDs showed lower lung function than patients with asthma without CTDs. With multivariable analysis, CTDs were independently associated with low FEV1 in asthma. Second, levels of markers for T2 inflammation were lower in asthma with CTDs.

Asthma subjects with CTDs showed lower FVC compared to those without CTDs. Interstitial lung diseases (ILDs) are often found with CTDs^16^ and might lead to lower FVC and FEV1 in asthma with CTDs. However, whether ILDs related to CTDs, affected FVC was unclear in this study, because not all asthma subjects underwent CT to check for ILDs. In addition, ILD was found in only 19.4% of subjects with CTDs (Table S2). A %FVC < 80% is considered a risk factor for asthma exacerbation^17^ and death in asthma subjects.^18^ Managing asthma with CTDs requires special attention due to the lower FVC. FEV1 was also lower in asthma with CTDs. Airway diseases such as BE or bronchitis are often found in patients with CTDs. For instance, one study found that 30%–40% of individuals with RA had BE.^19^ Airway diseases related to CTDs might result in a lower FEV_1_ in asthma with CTDs. In fact, Wisher et al. reported that 20% of RA patients showed an obstructive pattern on spirometry.^20^ In addition, asthma with CTDs required more intensive treatment than asthma without CTDs, as GINA step and the proportions of subjects using LABAs were higher in subjects with CTDs than in those without CTDs. This might be a result of the low FEV1 in asthma patients with CTDs. Low FEV1 is related to poor prognosis in both the general population and individuals with asthma.^21^, ^22^ In the presence of poor lung function, asthma with CTDs might be related to poor prognosis, but further study is needed to clarify this issue.

Peripheral eosinophils and total IgE were lower in asthma with CTDs. Peripheral eosinophils and high IgE level reflect T2 inflammation. On the other hand, T1 and T17 inflammation, rather than T2 inflammation, are typical in RA,^23^, ^24^ the most common CTD in this study. T1 and T17 inflammation are implicated in low‐T2 asthma.^25^ This might be related to a lower proportion of peripheral eosinophils and low IgE level in asthma with CTDs. Further study is needed to validate these findings.

Another point requiring attention in asthma with CTDs is immunosuppressive conditions in CTDs. Subjects with CTDs often receive systemic steroids, immunosuppressants and/or biologics, which result in varying degrees of immunosuppression. In fact, our previous study found that collagen vascular diseases were a risk factor for systemic corticosteroid use in asthma.^26^ Respiratory tract infection is the most common cause of asthma exacerbation,^27^ and prevention of such infections is important for asthma management. Moreover, asthma increases the risk of pneumonia.^28^ One preventive measure is vaccination against influenza and *Streptococcus pneumoniae*. As vaccination rates remain unsatisfactory even among the elderly with comorbidities^29^ or asthma,^30^ this measure should be further encouraged.

In this study, 27.8% (10/36) of individuals with asthma and CTDs displayed BE on chest CT. BE in asthma is related to deteriorated lung function and exacerbation of asthma.^31^ Whether BE is more frequent in asthma with CTDs remained unclear, because we had no control group. Further study is therefore warranted to clarify whether differences in the frequency of BE exist between asthma with CTDs and asthma without CTDs.

The aetiology of concomitant asthma and CTDs is under investigation. One cause is mucosal inflammation. Airway mucosal inflammation is related to the production of anti‐citrullinated protein antibodies.^32^ Zaccardelli et al. reported that asthma was associated with seropositive RA.^6^ In addition, asthma and some CTDs share genetic susceptibilities. Common genetic risks have been reported between asthma, RA and SLE.^33^, ^34^ Another key player causing concomitant asthma and CTDs might be T17 inflammation. T17 cytokines and inflammation have been implicated in the pathogenesis of a variety of CTDs.^23^, ^24^ T17 is also involved in asthma.^35^ Moreover, environmental factors such as smoking might also be relevant.^36^, ^37^

This study showed various limitations that should be considered. First, this was a retrospective study, and the diagnostic criteria for asthma were not predefined. In addition, not all subjects had data on peripheral eosinophils, IgE, lung function or CT within 2 years before and/or after the start of the study. However, asthma was diagnosed by an allergist and/or pulmonologist in all cases. About 90% of cases of asthma with CTDs underwent CT evaluation within 2 years before and/or after the start of the study. Second, diagnosis of asthma is challenging in subjects with CTDs, because some subjects with CTDs have obstructive lung function, which might be related to lung manifestations of CTDs. In addition, treatment for CTDs can affect asthma biomarkers. In this study, T2 inflammatory markers were lower in asthma with CTDs. However, some asthma patients with CTDs received systemic corticosteroids for the CTDs. Thus, low levels of Th2 inflammatory marker in asthma with CTDs may have been a result of therapeutic interventions. But the number of subjects receiving systemic steroids was low, it is unlikely to bias the results.

In conclusion, asthma with CTDs was related to low FEV1 and low‐T2 inflammation asthma. Further study is needed to clarify the clinical features of asthma with CTDs.

## AUTHOR CONTRIBUTIONS

Keisuke Watanabe contributed to the conception and design of the study; to the collection, analysis and interpretation of the data; and to drafting and finalizing the manuscript. Nobuyuki Horita, Yu Hara, Nobuaki Kobayashi and Takeshi Kaneko contributed to the interpretation of the data and to finalizing the manuscript. All authors reviewed the manuscript.

## CONFLICT OF INTEREST STATEMENT

K.W. has received research grants and/or personal fees from AstraZeneca, Novartis, Ono Pharmaceutical, Boehringer Ingelheim and Daiichi Sankyo outside of the present work. N.H. has received research grants and/or personal fees from Taiho Pharmaceutical outside of the present work. Y.H. has received research grants and/or personal fees from AstraZeneca, GlaxoSmithKline and Boehringer Ingelheim outside of the present work. N.K. has received research grants and/or personal fees from Chugai Pharmaceutical, AstraZeneca, GlaxoSmithKline, Boehringer Ingelheim, Pfizer, Sanofi, Ono Pharmaceutical, MSD, Bristol Myers Squibb, Eli Lilly, Daiichi Sankyo and Kyowa Kirin outside of the present work. T.K. has received research grants and/or personal fees from Chugai Pharmaceutical, AstraZeneca, Novartis, GlaxoSmithKline, Boehringer Ingelheim, KYORIN Pharmaceutical, Otsuka Pharmaceutical, Eli Lilly, Taiho Pharmaceutical, Sanofi, Pfizer, Shionogi and Teijin Pharma outside of the present work. This study was partially supported by research grants from Teijin Pharma. Teijin Pharma had no role in this study.

## ETHICS STATEMENT

This study was approved by the institutional review board of Yokohama City University Hospital (approval no. F210900006). Due to the retrospective nature of this study, the need to obtain written informed consent was waived by the institutional review board of Yokohama City University Hospital. All methods were carried out in accordance with relevant guidelines and regulations.

## Supporting information


**Table S1** Factors related to low FEV1 in asthma without COPD
**Table S2** Concomitant lung disease in asthma with CTD based on CT findings (n=36)

## Data Availability

The datasets used and/or analysed during the current study are available from the corresponding author on reasonable request.
