# Supplementary material for: Clinical features of asthma with connective tissue diseases
Source: Clin Respir J. 2023 Feb 18;17(4):303–10. doi: 10.1111/crj.13595 (PMC10113276; doi:10.1111/crj.13595)
Supplement: Supplementary file 1 — Table S1 Factors related to low FEV1 in asthma without COPD Table S2 Concomitant lung disease in asthma with CTD based on CT findings (n=36) [file CRJ-17-303-s001.docx]

**Supplementary**

Allergens associated with asthma are listed above (38).

Cockroach, Dander (cat, dog), House dust mite (*Dermatophagoides pteronyssinus*, *Dermatophagoides farina*), Mold spores (*Alternaria, Cladosporium, Helminthosporium, Aspergillus, Penicillium*) and Pollens (reginal tree, grass, and weed pollen).

Table s1 Factors related to low FEV1 in asthma without COPD

|  | Asthma with low FEV1  (n=88) | Asthma without low FEV1  (n=208) | P | |
| --- | --- | --- | --- | --- |
|  |  |  | univariate analysis | multivariable analysis |
| Age (years) | 61 (22-84) | 60 (21-88) | 0.63 |  |
| Sex (male/female) | 31/57 | 68/140 | 0.69 |  |
| Smoking history  (never/ex/current) | 65/16/6  (n=87) | 135/49/23  (n=207) | 0.26 |  |
| Onset at <20 years old | 20 (24.4%)  (n=82) | 37 (19.0%)  (n=195) | 0.33 |  |
| Allergic asthma | 43 (48.9%) | 90 (43.3%) | 0.44 |  |
| CTDs | 14 (15.9%) | 16 (7.7%) | 0.04 | 0.03 |
| AR | 22 (25.0%) | 53 (25.5%) | 1 |  |
| Sinusitis | 8 (9.1%) | 17 (8.2%) | 0.82 |  |

Data are presented as median (range) or number of patients (%).

Low FEV1 is defined as percentage predicted forced expiratory volume in 1 s (%FEV1) <80%.

AR, allergic rhinitis; COPD, chronic obstructive pulmonary disease; CTDs, connective tissue diseases

Table s2 Concomitant lung disease in asthma with CTD based on CT findings (n=36)

| Bronchiolectasis | 10 (27.8%) |
| --- | --- |
| Emphysema | 8 (22.2%) |
| Interstitial lung disease | 7 (19.4%) |
| Cystic lesions | 2 (5.6%) |
| Pleuritis | 0 (0%) |
